# Biomarker discovery of disrupted SORL1‐retromer recycling

**DOI:** 10.1002/alz.084837

**Published:** 2025-01-03

**Authors:** Scott A. Small

**Affiliations:** ^1^ Columbia University Irving Medical Center, New York, NY USA

## Abstract

Biofluidic biomarkers concord with postmortem molecular studies, suggesting that the endosomal recycling pathway regulated by SORL1’s interaction with the retromer protein VPS2b is commonly disrupted in late‐onset, ‘sporadic’, Alzheimer’s disease (AD). Here, a program for developing a neuroimaging‐based biomarker will be reviewed. The program is anchored by findings in support of the conclusion that, because of its distinct network properties, the trans‐entorhinal cortex is heavily dependent on the recycling pathway. Functional and structural MRI have documented that the trans‐entorhinal cortex is commonly affected in AD, and ex vivo studies have found that this AD‐targeted region is co‐deficient in SORL1 and VPS26b. Since SORL1 deficiency is considered causally pathogenic in AD, we have applied our dedicated small‐animal MRI to longitudinally image SORL1 haploinsufficient mice. Remarkably, results show that SORL1 haploinsufficient mice developed MRI‐detected focal atrophy in the trans‐entorhinal cortex at 12 months of age, and which corresponded to the observed spatiotemporal pattern of SORL1 deficiency. Next, we used viral vectors to overexpress VPS26b, at 3‐month‐old mice and aged them to 12 months. Replicating the primary finding, untreated 12‐month‐old SORL1 haploinsufficient mice showed selective trans‐entorhinal cortex atrophy and SORL1 deficiency. AAV9‐VPS26b treatment was found to prevent the development of MRI‐detected atrophy. Collectively, these studies support the conclusion that MRI‐detected trans‐entorhinal cortical atrophy might represent an imaging‐based biomarker of pathway dysfunction. We are currently developing an MRI template of the AD‐targeted trans‐entorhinal cortex and plan to apply to it to future large‐scale datasets of common and rare forms of AD.

